# Implementation of the International Code of Marketing of Breast-milk Substitutes and maternity protection: correlations with commercial milk formula consumption in East Asia and the Pacific

**DOI:** 10.3389/fped.2025.1553599

**Published:** 2025-07-09

**Authors:** Constance Ching, Tuan T. Nguyen, Catherine Pereira-Kotze, Paul Zambrano, Phillip Baker, Roger Mathisen

**Affiliations:** ^1^ Alive & Thrive, FHI 360 Global Nutrition, Hanoi, Vietnam; ^2^ College of Health Sciences, VinUniversity, Hanoi, Vietnam; ^3^ Alive & Thrive, FHI 360 Global Nutrition, Washington, DC, United States; ^4^ Alive & Thrive, FHI 360 Global Nutrition, Manila, Philippines; ^5^ School of Public Health, University of Sydney, Sydney, NSW, Australia

**Keywords:** breastfeeding, breastmilk substitutes (BMS), commercial milk formula (CMF), CMF consumption, CMF sales, International Code of Marketing of Breast-milk Substitutes (the Code), maternity protection, East Asia Pacific

## Abstract

**Objective:**

The rise of commercial milk formulas (CMF) consumption by infants and young children raises concerns about their health, development, and survival. Implementing the International Code of Marketing of Breast-milk Substitutes (the Code) and maternity protection policies are two of seven critical policy actions to protect breastfeeding. This study explores the implementation of the Code, maternity protection policies, and CMF consumption in 11 countries in the East Asia Pacific (EAP) region and determines whether there are any correlations.

**Methods:**

Data on CMF consumption (from 2006 to 2019 with the projection to 2024 at the global, regional, and national levels), Code implementation scores and the age range covered by national measures, and maternity protection policies were used. Simple linear regressions were conducted to explore correlations between CMF consumption and Code implementation as well as maternity protection.

**Findings:**

In 2019, EAP contributed to 63% of global CMF consumption, toddler formula was the highest category, a product that is unnecessary and unsuitable for consumption by young children. Sales volumes and per capita consumption of most CMF types have increased in the region between 2006 and 2024. Currently, nine out of the 11 countries have adopted Code legal measures. Japan and Malaysia have none and are relying on voluntary measures. Australia, New Zealand, and Singapore now have some Code provisions as legal measures (none in 2020). CMF marketing in Australia and New Zealand remain largely voluntary. Only the Philippines and Thailand are covering CMF up to 36 months. Seven of the 11 countries provide the minimum maternity protection entitlements based on International Labour Organization (ILO) standards. As total scores on Code implementation increased, per capita CMF consumption decreased. Most countries with high overall CMF consumption had no Code legal measures. Philippines, with the highest Code implementation score, showed the lowest per capita CMF consumption. Though no correlation found between CMF consumption and paid maternity leave duration, other forms of maternity protection were not included in the regression.

**Recommendation:**

Adopt legal measures to give full effect to the Code as opposed to relying on voluntary measures. Prioritise strengthening comprehensive maternity protection, cultivate intersectoral policy environments.

## 1 Introduction

In the East Asia and Pacific (EAP) region, there are approximately 174,394 neonatal deaths (median) each year (1). Additionally, 18.8 million children experience stunting, 4.9 million suffer from wasting, and 10.9 million are overweight (2). Improving infant and young child feeding (IYCF) practices, specifically early initiation of breastfeeding, exclusive breastfeeding for the first six months, and continued breastfeeding for at least two years with appropriate complementary feeding, can significantly reduce child mortality and morbidity (3). Yet only 40% of newborns are put to the breast within one hour of birth (early initiation), 42% of infants are exclusively breastfed for the first six months of life, and only 39% of children 12–24 months of age are continuously breastfed in the EAP region (4). Not protecting, supporting and promoting breastfeeding in line with recommendations from the World Health Organization (WHO) and United Nations Children's Fund (UNICEF) is associated with high mortality, morbidity, and health system expenses. As a result, close to 67,000 lives (due to associated with child diarrhea, acute respiratory illness, and obesity; and adult breast cancer, ovarian cancer, and type II diabetes), over 17,300,000 school years, approximately 49,700,000 IQ points, and over US$145 billion are lost due to suboptimal breastfeeding in the EAP region per year (5, 6). Milk provided by breastfeeding mothers is a crucial national food resource which also reduces water and carbon footprints substantially (7). However, it remains largely invisible, and its economic value remains unmeasured (8). Globally, suboptimal breastfeeding means that 38.2% of breastmilk is currently “lost”, contributing to US$ 2.2 trillion of loss in value annually (9).

Breastfeeding is influenced by multiple factors at the structural, setting, and individual levels - spanning from legal and policy directives to socioeconomic conditions, health care system, marketing practices, social and family support, and workplace maternity protection policies, social support network, breastfeeding knowledge and attitudes, and health (10). The pervasive and unethical marketing of commercial milk formula (CMF) for infants and young children [otherwise known as breastmilk substitutes (BMS)] and related products and inadequate maternity protection remain two of the major barriers to scaling up breastfeeding (11–13).

### 1.1 Unethical marketing and the International Code

Exposure to the marketing of CMF and related products has been linked to a reduction in initiation, exclusivity, and duration of breastfeeding (14–18). Through advertising and promotion, funding and sponsorship, political lobbying, research, interest group pressure, and even economic threats, the CMF industry influences members of the public, health professionals, governments, and politicians at different levels to directly and indirectly increase sales and maximise profits (13, 19, 20). In the last two decades, the CMF industry has also taken advantage of the viral and personalised nature of digital marketing strategies to amplify promotion, such as planting targeted advertisements on pregnant women's and mothers’ cell phone applications, enticing clandestine participation in online clubs and forums, or persuading mothers to market CMF using their own content or social media networks (21).

The World Health Assembly (WHA) adopted the International Code of Marketing of Breast-milk Substitutes in 1981 (22). Together with subsequent relevant WHA resolutions, (collectively known as “the Code” hereinafter), it aims to restrict all forms of promotion that undermine breastfeeding and inappropriate IYCF by outlining responsibilities of governments, health systems, health workers, and CMF companies. It also serves to protect infants and young children who use CMF by requiring information on health hazards, appropriate use, and safe preparation (23) (See Supplementary Tables S1 and S2 for summary and scope of the International Code and relevant WHA resolutions). In 2023, WHO published the *Guidance on Regulatory Measures aimed at Restricting Digital Marketing of Breast-milk Substitutes* (the 2023 Guidance) to provide recommendations to governments specifically on developing and applying regulatory measures aimed at restricting digital marketing of products that fall within the scope of the Code and foods for infants and young children (24). Governments are obligated to adopt the Code in full in the form of national legal measures, as these marketing practices undermine the right of the child to the highest attainable standard of health as affirmed in the United Nations (UN) Convention on the Rights of the Child (CRC) (25).

Even though a number of countries in this region have adopted legal measures to give effect to the Code, ongoing inappropriate marketing that violates the Code and national laws in this region has been frequently reported (26–32).

### 1.2 Rapid growth in CMF markets

CMF (BMS) global sales have increased two-folds in the past two decades, reaching US$55.6 billion per year in 2019 (33). Even though the WHO has long maintained that follow-on formula and toddler formula are not necessary and unsuitable as substitutes for continued breastfeeding (34), these two categories now comprise the majority of global CMF sales (35, 36). In the Asia Pacific region, where much of the market growth occurred, the market size has almost doubled from US$18.7 billion to US$36.4 billion, and from comprising 56% of global sales to 68% in just ten years (from 2010 to 2020) (37). During the same period, Asia Pacific also experienced the highest proportional growth in annual per capita consumption on CMF (77%: US$4.83 to US$8.53) out of all global regions (constant 2020 prices and fixed exchange rates) (37). While demand side factors such as income growth are important, these increases suggest commercial determinants, particularly the CMF industry's marketing strategies, have been effective in generating CMF consumption and displacing breastfeeding (13, 33–36).

### 1.3 Maternity protection

Apart from Code implementation to regulate marketing, another important determinant of breastfeeding is maternity protection (19). Comprehensive maternity protection includes a set of entitlements for working women who are pregnant, around the time of childbirth and while breastfeeding, such as health protection at the workplace, maternity leave with accompanying cash and medical entitlements, job security, non-discrimination and breastfeeding or expressing breaks (38, 39). In low-and-middle-income countries (LMIC), extending the duration of legislated paid maternity leave is associated with improved breastfeeding practices (40), and reduced infant mortality (41). Research from high-income countries shows improved maternal health outcomes and paternal mental health with more comprehensive parental leave policies (42, 43). Even though many countries do meet some of the minimum global standards for paid maternity leave (44), very few countries, and none in Asia, have formally ratified the Maternity Protection Convention (45). Furthermore, the availability of maternity protection does not guarantee its access. For example, research from South Africa reveals that while domestic workers (a particularly vulnerable group of informal workers) have had legal entitlements to social insurance for the past 20 years, which enable cash payments to be available while on maternity leave, very few workers can access these entitlements due to complex implementation challenges (46). In Asia, while it is encouraging that between 2010 and 2021, nine countries in Asia increased maternity leave to at least 14 weeks (44), it is concerning that 94.4% of women in Asia-Pacific live in countries with no mandatory maternity cash payments for self-employed workers. More than 50% of working women in Asia-Pacific work informally (47), where incomes are often low and unpredictable and employment relationships are precarious. Without mandatory cash payments, many women cannot access maternity leave entitlements, will need to return to work early and not be able to afford to use the maternity leave available to them, and the likelihood of breastfeeding will decrease.

### 1.4 Relevant studies and gaps

A 2013 study, using data on breastfeeding duration and World Breastfeeding Trends Initiative (WBTi) scores in 22 countries across regions, suggested the possibility of measuring the causal relationship between breastfeeding protection, promotion, and support and improved breastfeeding practices (48). Another study in 2015 contrasted CMF sales with information on Code implementation and breastfeeding practices between China and India. The study also acknowledged that factors other than Code implementation, including women's labour force participation rate and income levels, are likely to influence CMF sales (49).

A recent study which examined the status of Code implementation in eight countries in South Asia (Afghanistan, Bangladesh, Bhutan, India, Maldives, Nepal, Pakistan, and Sri Lanka) found major gaps in Code-related legal measures (as compared to the Code) and a lack of monitoring and enforcement across the region, even in countries with strong legal measures (50). The same study also explored the potential correlations between Code implementation on CMF and baby food sales value and volume of four of those countries (Bangladesh, India, Pakistan, and Sri Lanka). A general pattern on sales and the scope of products covered in national legal measures was observed: Infant formula, a CMF product marketed for infants from birth to six or 12 months and most commonly covered in the scope of national legal measures in the region, sustained stagnant sales growth compared to products marketed for older infants and young children. However, several gaps and limitations persist - the observation was non-statistical, and other factors that may have influenced breastfeeding practices and sales of CMF, including maternity protection, were not assessed.

### 1.5 Aim of study

This study seeks to adapt from the 2023 South Asia study discussed above (50) in 11 countries in the East Asia Pacific region, (Australia, China, Indonesia, Japan, Malaysia, New Zealand, the Philippines, Singapore, South Korea, Thailand, and Vietnam), aiming to:.

• Examine data on Code implementation status, maternity protection policy, and CMF consumption in the 11 countries
• Explore whether there are correlations between Code implementation (marketing regulations on CMF) and CMF consumption
• Explore whether there are correlations between maternity protection and CMF consumption

## 2 Methodology

### 2.1 Study setting

The 11 countries were selected based on availability of and access to CMF consumption data in the East Asia Pacific region. They are from four income groups, namely low, lower-middle, upper-middle, and high income, classified using the World Bank Atlas method that converts the gross national income (GNI) per capita data from local currency to USD (51). The Philippines and Vietnam are classified as lower-middle-income. China, Indonesia, Malaysia, and Thailand are classified as upper-middle-income. Five of the 11 countries are high-income: Australia, Japan, New Zealand, Singapore, and South Korea.

### 2.2 Assessment on Code implementation

Data on the Code implementation status of the 11 countries was extracted from the 2020 and 2024 WHO/UNICEF/IBFAN Marketing of breast-milk substitutes: National implementation of the International Code Status Reports (hereinafter known as “the 2020 and 2024 WHO Code Status Reports”) (52, 53). The Code implementation status was assessed based on the extent to which the provisions of the Code and relevant WHA resolutions have been incorporated in the national legal measures in seven areas: Scope; monitoring and enforcement; informational/educational materials on IYCF; promotion to the general public; promotion in health facilities; engagement with health workers and systems; and labelling. The reports used a scoring algorithm (total maximum score at 100) to reflect the national status of overall Code implementation. The total scores are then ranked in four categories:

• Substantially aligned with “the Code”: Score of 75–100
• Moderately aligned with “the Code”: Score of 50–<75
• Some provisions of “the Code”: Score of <50
• No legal measures (includes countries that have drafted legislation but not enacted it)

The Code Status Reports also indicate the range of products covered at the national level, including the age range (in months) of CMF covered in national legal measures, using the benchmark of 36 months as recommended by the 2016 Guidance that was part of the WHA Resolution 69.9 (54, 55) (hereinafter known as the “2016 Guidance”). This study used two indicators to reflect on the status of Code implementation: (1) The total Code implementation scores, and (2) the age range of CMF products covered in national legal measures.

It is important to highlight that, apart from CMF products, the Code also covers marketing of bottles and teats and foods for infants and young children. However, this study focused on CMF products due to the limited access to sales data.

### 2.3 Maternity protection

Data on maternity leave duration (in weeks) and cash entitlements [amount, source, and alignment with the ILO Maternity Protection Convention (MPC) 183], as well as data on breastfeeding breaks (such as whether breastfeeding breaks are provided, duration, and whether they are paid) of the 11 countries was obtained from the 2022 ILO *Care at work: Investing in care leave and services for a more gender equal world of work* report (44). The report surveyed 185 countries, including the 11 countries included in this study.

### 2.4 CMF consumption

Data on the global, regional, and national CMF consumption between 2005 and 2019, with predictions to 2024, was obtained from Euromonitor, a data analytics company that offers market reports and forecasts through a license with Deakin University (56). Related data was extracted from Euromonitor using its pivot function to generate Microsoft Excel spreadsheets, with the key indicators of sales volumes and per capita consumption (sales volumes divided by the number of children in the age category) on CMF by the CMF category, year, and country.

The CMF products included in the data are classified into:

• Standard formula: Otherwise known as “infant formula”, “first stage formula”, or “starter formula”, these are formula products marketed for babies from birth. The figure “1” is normally used on the label.
• Follow-on formula: Otherwise known as “follow-up formula” or “second stage formula”, these are formula products commonly marketed for babies from 6 months of age and above. The upper age indication varies from country to country but is usually between 12 and 24 months. The figure “2” is normally used on the label.
• Toddler formula: Otherwise known as “growing-up milk” or “third-stage formula”, these are formula products commonly promoted for young children between 1 and 3 years of age. The figure “3” is usually used on the label.
• Special formula: These products usually include soy formula, lactose-free formula, and low birthweight/premature formula marketed for infants with specific medical conditions, diseases, or disorders. Their use is usually recommended by medical prescription or advice.

Microsoft Excel PivotTables and figures were used to extract relevant findings from the Excel spreadsheet to generate relevant tables and figures.

Regional and country-level trends in EAP were reviewed, and the values of countries were compared with sales volumes data in the EAP region. A similar analysis for the per capita consumption on CMF was performed.

### 2.5 Exploring correlations between Code implementation and CMF consumption, and between maternity protection and CMF consumption

Simple linear regression analyses using Microsoft Excel were used to explore the potential correlations between three pairs of continuous variables. Since the analyses were exploratory, pre-defined hypotheses were not used. The analyses sought to explore:

• The total scores on Code implementation and per capita consumption on CMF by type and country
• The age (in months) of which CMF products are covered under the national legal measures and per capita consumption by CMF type and country
• The duration of paid maternity leave and per capita consumption on CMF by type and country

The total Code implementation scores used in exploring correlations are extracted from the 2020 Code Status Report (52) instead of the latest 2024 report (53), because the 2019 data on CMF consumption was used in this study. Using the 2020 Code implementation scores is to ensure the data on Code implementation and CMF consumption have the closest timepoints possible.

## 3 Results

### 3.1 Implementation of Code provisions in national legal measures

Among the 11 countries included in this study, there are varying extents of implementation of Code provisions in legal measures (Table 1). In 2020, six countries (China, Indonesia, the Philippines, South Korea, Thailand, and Vietnam) adopted some legal measures to implement the Code. The total implementation scores of each country ranged from 25/100 (some provisions of the Code included) to 85/100 (substantially aligned with the Code). With a total score of 85/100, the Philippines enacted legal measures encompassing a significant set of provisions of the Code, making it substantially aligned with the Code (category with scores of 75/100–100/100). Three out of the six countries, Indonesia, Thailand, and Vietnam, with total scores of 50/100, 68/100 and 73/100, respectively, had legal measures that were moderately aligned with the Code by encompassing a majority of provisions of the Code (category with scores of 50/100–<75/100). China was in the category “some provisions of the Code included”, meaning it only included less than half of the provisions outlined in the Code and relevant WHA resolutions (category with scores of <50/100). Australia, Japan, Malaysia, New Zealand, and Singapore had no legal measures to give effect to the Code.

**Table 1:** Total scores, code implementation status, and scope of commercial milk formula covered, extracted from the 2020 and 2024 code Status reports (52, 53).

Year		Australia	China	Indonesia	Japan	Malaysia	New Zealand	Philippines	Singapore	South Korea	Thailand	Vietnam
2020	Total score (0- of 100)	No legal measures	25	50	No legal measures	No legal measures	No legal measures	85	No legal measures	26	68	73
	Status of implementation	No legal measures	Some provisions of the Code including	Moderately aligned with the Code	No legal measures	No legal measures	No legal measures	Substantially aligned with the Code	No legal measures	Some provisions of the Code included	Moderately aligned with the Code	Moderately aligned with the Code
	BMS products covered up to age (months)	N/A	12	12	N/A	N/A	N/A	36	N/A	Unspecified	36	24
2024	Total score (0- 100)	27	27	63	No legal measures	No legal measures	27	85	27	26	65	79
	Status of implementation	Some provisions of the Code included	Some provisions of the Code included	Moderately aligned with the Code	No legal measures	No legal measures	Some provisions of the Code included	Substantially aligned with the Code	Some provisions of the Code included	Some provisions of the Code included	Moderately aligned with the Code	Substantially aligned with the Code
	BMS products covered up to age (months)	12	12	Unspecified	N/A	N/A	12	36	12	Unspecified	36	24

BMS, breastmilk substitutes; N/A, not applicable due to no legal measures, it was assigned zero in the correlation analysis.

As of 2024, Australia, New Zealand, and Singapore have moved from “no legal measures” to “some provisions of the Code included” (where they were recorded as having none in 2020, according to the 2020 Code Status Report). The food regulations of Singapore underwent a review and now include several Code-related provisions, making the country's status as having enacted some Code provisions (27/100). Australia and New Zealand have also adopted some labelling provisions of the Code in their Food Standards Act, but marketing is still restricted through non-legally binding agreements (27/100 for both countries). Together with China, Indonesia, the Philippines, South Korea, Thailand, and Vietnam, a total of nine countries out of the 11 have legally binding measures to give effect to the Code, with total scores ranging from 26/100 (some provisions of the Code included) to 85/100 (substantially aligned with the Code). Japan and Malaysia have no legal measures to give effect to the Code (voluntary measures), hence there was no score allocated for these two countries.

The Philippines and Vietnam, with total scores of 85/100 and 79/100, respectively, are substantially aligned with the Code, as they have enacted legislation or adopted regulations encompassing a significant set of provisions of the Code (score of 75/100–100/100). Indonesia and Thailand, with total scores of 63/100 and 65/100, respectively, have legal measures that are moderately aligned with the Code, encompassing a majority of provisions of the Code (score of 50/100–<75/100). Australia (27/100), China (27/100), New Zealand (27/100), Singapore (27/100), and South Korea (26/100) all have legal measures that include some provisions of the Code, covering less than half of the provisions outlined in the Code and relevant WHA resolutions (score of <50/100). Vietnam has moved from “moderately aligned with the Code” in 2020 to “substantially aligned with the Code” in 2024 as it issued a new decree in 2021 to update and revise the sanctions that can be applied for enforcement.

### 3.2 Scope of CMF products covered in national measures

As of 2020, among the six countries that have adopted legal measures to give effect to the Code, only the Philippines and Thailand covered CMF products up to 36 months of age in the scope, as recommended by the 2016 Guidance. Vietnam covered CMF products up to 24 months, leaving gaps for promotion of toddler milk products. China and Indonesia only covered CMF products up to 12 months (common age duration for standard formula and special formula), leaving gaps for promotion of follow-on formula and toddler formula. South Korea's scope was “unspecified”, leaving it difficult for overall implementation. Australia, Japan, Malaysia, New Zealand, and Singapore had no legal measures to give effect to the Code, thus the definition of scope is considered not applicable in this context.

In 2024, with Australia, New Zealand, and Singapore having adopted some Code provisions, the scope of CMF products in their national measures changed to 12 months. Together with China, these four countries have legal measures that apply to CMF products marketed as suitable for up to 12 months, which is the cut-off age of most standard and special formula products, presenting large gaps when compared to the scope of 36 months recommended in the 2016 WHO Guidance.

Although the Philippines and Vietnam are the only two countries with total scores that are substantially aligned with the Code, the scope of CMF of the Philippines is up to 36 months, covering the promotion of CMF including standard formula, special formula, follow-on formula, and toddler formula. This also applies to Thailand, meaning the scope of both the Philippines and Thailand is aligned with the 2016 WHO Guidance. Whereas in Vietnam, the scope is only up to 24 months, leaving a gap for the promotion of toddler formula.

Indonesia and South Korea have adopted legal measures to give effect to the Code, however the age range to which the measures apply regarding the marketing of CMF products is unspecified, leaving it close to impossible for monitoring and enforcement to take place.

### 3.3 Maternity protection

Seven of the 11 countries are aligned with the ILO MPC (Australia, China, Japan, New Zealand, the Philippines, Singapore, and Vietnam), with New Zealand and Vietnam providing six months of paid maternity leave. Various configurations of paid maternity leave exist, including payment at 100% of previous earnings for the full duration of maternity leave (Indonesia, Vietnam, Malaysia and China), 100% up to a ceiling (New Zealand), 100% for part of the leave and a lower rate for the remainder (Thailand, South Korea), 100% for the first two children (Singapore), and payment at the national minimum wage. Other variations include partial payment, as in Japan's case (66.7% of previous earnings). It should be acknowledged that some women may not be able to use their full maternity leave entitlement for times when it is not paid at 100% of previous earnings. Australia, China, Japan, Singapore, and the Philippines provide 14–19 weeks of paid maternity leave. Of the four countries that are not aligned with the ILO MPC (Indonesia, Malaysia, South Korea, and Thailand), South Korea, Indonesia and Thailand all provide 13 weeks, and Malaysia provides the lowest at 9 weeks of maternity leave (Table 2).

**Table 2:** Maternity protection in East Asia Pacific region.

Characteristics related to maternity protection	Country										
	Australia	China	Indonesia	Japan	Malaysia	New Zealand	Philippines	Singapore	South Korea	Thailand	Vietnam
Labour force participation:											
Number of adults aged 15–64 years (million) (2022)[1]	16.9	974.8	187.2	73.1	23.7	3.3	74.2	4.1	36.6	49.7	67.2
Labour force participation rate^[2]^, males (% of population ages 15+) (2023)^[3]^	71.4	72.1	81.9	71.5	78.0	76.8	73.1	76.2	73.3	75.6	77.8
Labour force participation rate, females (% of population aged 15+) female (2023)^[4]^	61.5	60.5	53.3	54.9	51.6	67.6	47.2	61.6	55.8	59.2	68.5
% in the formal sector											
% in informal sector^[5]^	26.1 (2022)	–	80.2 (2022)	–	–	–	–	–	26.6 (2019)	65 (2018)	68.6 (2022)
% vulnerable employment male (2022)^[6]^	12.3	43.0	44.5	8.5	18.8	21.9	30.0	12.0	19.7	49.4	46.9
% vulnerable employment female (2022)^[7]^	8.2	40.8	59.3	7.4	24.3	16.1	38.5	6.6	17.1	50.1	57.3
Paid maternity leave^[8]^											
Maternity leave duration (weeks)	52	14	13	14	8.5	26	15	16	13	13	26
Cash entitlements: amount	740 AUD/wk	100%	100%	66.7%	100%	100% but up to 585.80 NZD	100%	100%	100% for 60 days, 100% up to 2 000 000 won for last 30 days	100% for 45 days, 50% for last 45 days	100%
Cash entitlements: source											
Social insurance		X		X			X		X	X	X
Employer liability			X		X		X	X			
Non-contributory scheme	X					X		X			
Provision of maternity leave cash benefits for self-employed workers	Yes	Yes (voluntary)	No	No	No	Yes	Yes	Yes	Yes	Yes	No
Aligned with Maternity Protection Convention No. 183	Yes	Yes	No	Yes	No	Yes	Yes	Yes	No	No	Yes
Breastfeeding breaks											
Breastfeeding break provided	No	Yes	Yes	Yes	No	Yes	Yes	No	Yes	No	Yes
Breastfeeding break duration (min)	–	60	60	60	–	Not specified^a^	40	–	60	–	60^[9]^
Breastfeeding break paid	–	Yes	Yes	Yes	–	No	Yes	–	Yes	–	Yes

^a^ These breaks are typically unpaid and should be of reasonable duration, depending on the mother's needs and the workplace's practicalities.

[1] https://data.worldbank.org/indicator/SP.POP.1564.TO.

[2] The labour force participation rate is the proportion of the population ages 15 and older that is economically active.

[3] https://genderdata.worldbank.org/.

[4] https://genderdata.worldbank.org/.

[5] https://ilostat.ilo.org/data/country-profiles/.

[6] https://genderdata.worldbank.org/.

[7] https://genderdata.worldbank.org/.

[8] ILO. 2022. Care at work: Investing in care leave and services for a more gender equal world of work. https://www.ilo.org/global/topics/care-economy/WCMS_838653/lang–en/index.htm.

[9] Mandatory at workplace ≥1,000 female employees.

Seven countries (China, Indonesia, Japan, New Zealand, the Philippines, South Korea, and Vietnam) provide breastfeeding breaks at the workplace; all are paid except for New Zealand. Breastfeeding breaks are provided for up to 60 min in four of these countries (China, Japan, South Korea, and Vietnam), with the Philippines providing 40 min (Table 2).

### 3.4 CMF sales volumes and consumption in EAP region

**Figure 1:**
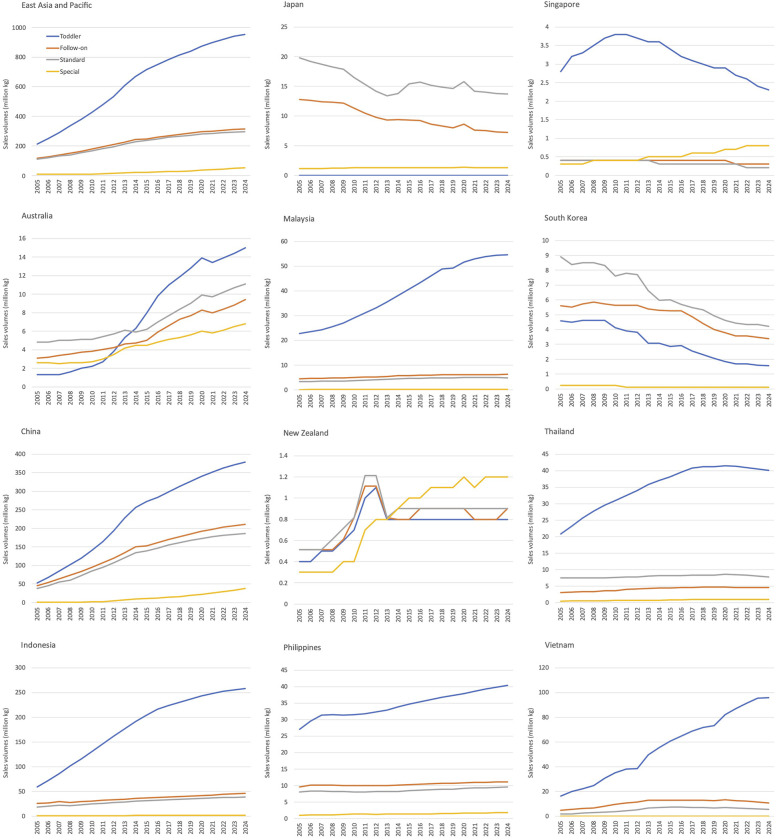
Sales volumes (million kg) of commercial milk formula in the East Asia Pacific region by type and country.

In the EAP region, the sales of CMF witnessed a notable increase, rising from 446.3 million kg in 2005 to 1.16 billion kg in 2014, 1.48 billion kg in 2019, and is projected to reach 1.62 billion kg in 2024 (data not shown). In EAP region, absolute sales volumes were evident across all categories of CMF, especially toddler formula (Figure 1). Certain CMF categories in Japan (follow on and special), Singapore (standard and toddler), and South Korea (all four categories) had decreased trend of sales volumes. The remaining categories of these three countries and other eight countries exhibited an increased or steady trend in CMF sales volumes across most categories (Figure 1). Australia and China showed substantial increased trend in all four categories of CMF. Except for Japan, New Zealand, and South Korea, toddler formula had the highest sales volumes compared to other CMF types (Figure 1). Additionally, Singapore, New Zealand, and South Korea observed a decreased trend of sales volumes (Figure 1).

In the EAP region in 2019, China and Indonesia ranked as the top two countries with corresponding sales volumes of 698.4 and 313.3 million kg, respectively. Markets with sales volumes ranging from 50 to 100 million kg include Vietnam, Malaysia, the Philippines, and Thailand, while those with sales volumes under 50 million kg include Australia, Japan, South Korea, Singapore, and New Zealand (Data not shown).

**Figure 2:**
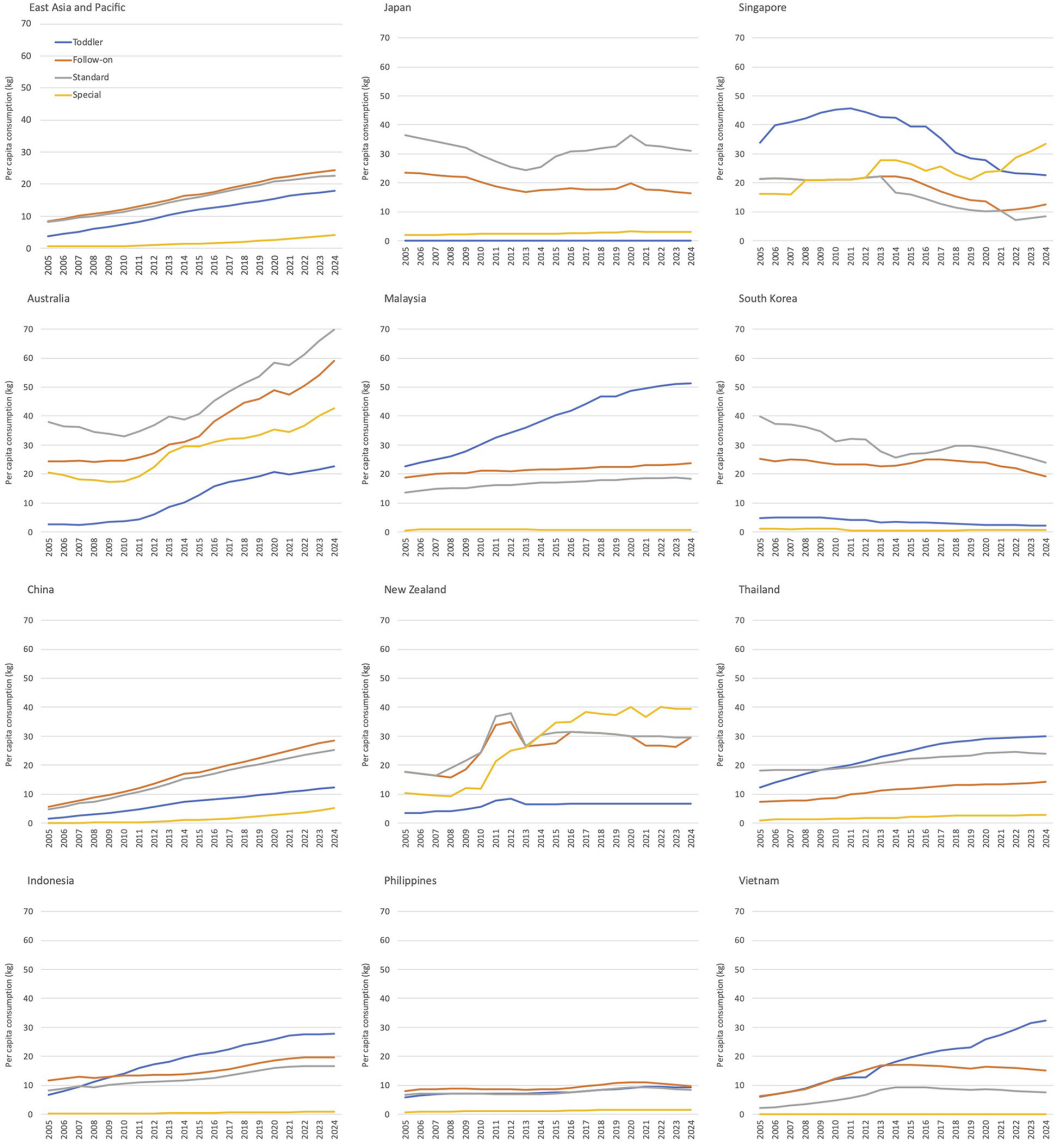
Per child consumption of commercial milk formula categories in the East Asia Pacific region by type and country.

The EAP region exhibited a high and increasing trend in annual per capita consumption across all CMF categories, with the follow-on formula category showing the highest expenditure, followed by standard, toddler, and special formulas (Figure 2). Countries exhibiting overall increases in per capita consumption were Australia and China (four CMF categories); Indonesia, Malaysia, and Thailand (three CMF categories); New Zealand, Singapore and Vietnam (one category) (Figure 2). Furthermore, toddler formula emerges as the highest per capita consumption in Indonesia, Malaysia, Singapore, Thailand, and Vietnam (Figure 2).

### 3.5 Correlations between Code implementation and per capita consumption of CMF

#### 3.5.1 Code implementation scores and per capita consumption of CMF

**Figure 3:**
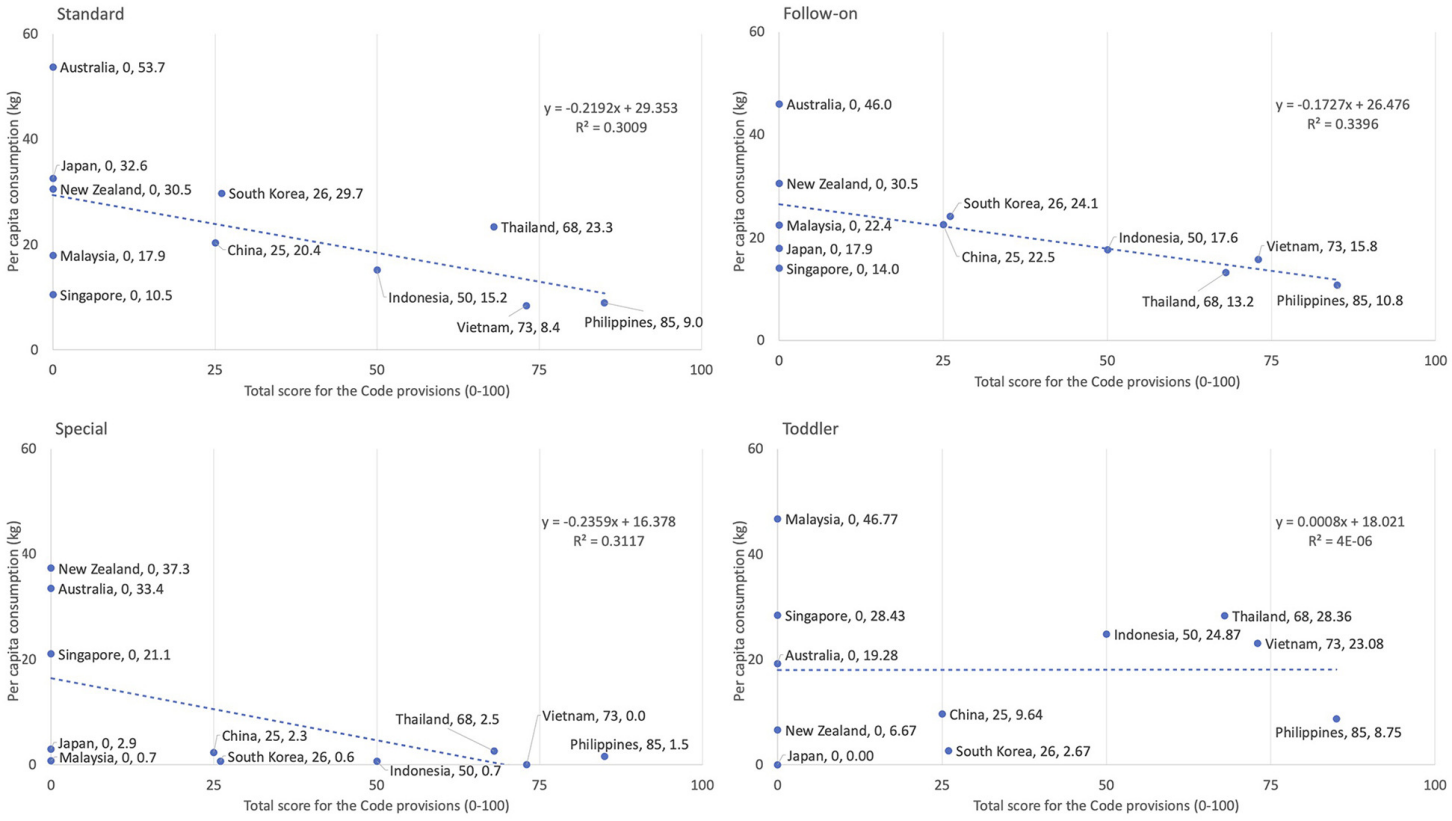
Association between the total scores (1–100) of Code provisions and per capita consumption of commercial milk formula in 2019 in East Asia Pacific region by type and country. In countries without any legal measures, or where legal measures exist but the value isn't specified a score of zero was assigned.

Figure 3 illustrates the correlations between Code implementation scores in 2020 (ranged 0–100) and per child consumption on CMF across the four categories: Standard, follow-on, toddler, and special formulas. For standard, follow-on, and special formulas, which are marketed for infants from birth to 12 or 24 months (depending on the brand and company), an overall inverse correlation with Code implementation scores was evident.

Countries with no legal measures to restrict marketing of CMF (zero Code implementation scores in 2020) had higher per capita consumption of standard formula, a product marketed to infants from birth (Australia at 53.7 kg, Japan at 32.6 kg, and New Zealand at 30.5 kg; as well as follow-on formula, a product usually marketed as suitable from six to 12 or 24 months (Australia at 46.0 kg and New Zealand at 30.5 kg) (Figure 3).

Comparatively, countries with stronger Code implementation to restrict marketing, such as Vietnam and the Philippines, which scored 73/100 and 85/100 respectively, had lower per capita consumption of standard formula (the Philippines at 9.0 kg and Vietnam at 8.4 kg); as well as follow-on formula, the Philippines at 10.8 kg and Vietnam at 15.8 kg. Other countries with lower to moderate scores such as China (25/100), Indonesia (50/100), Korea (26/100), and Thailand (68/100) had intermediate levels of consumptions (Figure 3).

The correlation (*R*^2^) was about 0.3 for follow-on, special, and standard formulas, and was about zero for toddler formula (Figure 3).

#### 3.5.2 Age of products covered in national legal measures and per capita consumption of CMF

**Figure 4:**
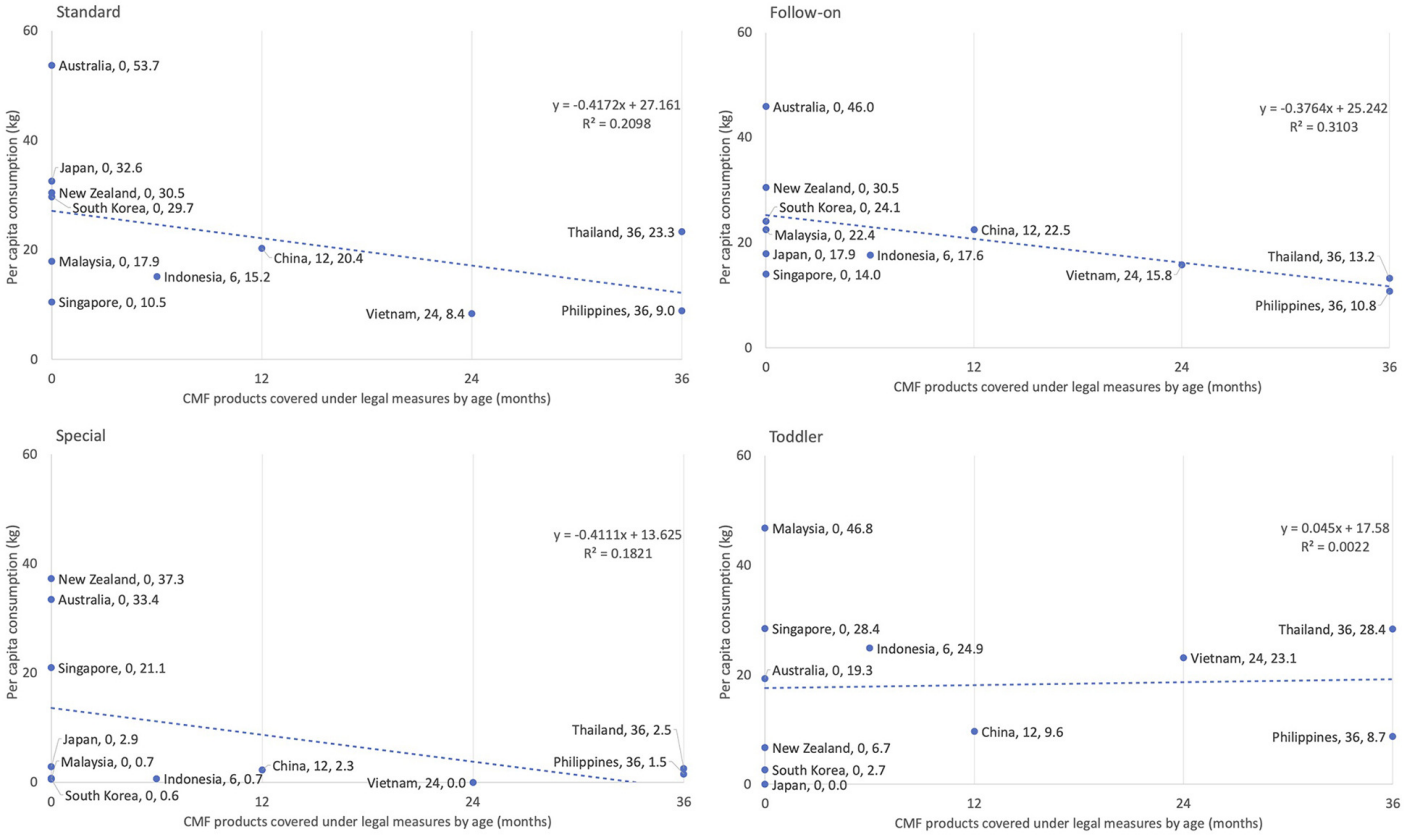
Association between the age in months at which CMF products are covered under the code and per capita consumption of commercial milk formula in 2019 by type and country in East Asia Pacific region. In countries without any legal measures, or where legal measures exist but the value isn't specified, a score of zero was assigned.

Figure 4 illustrates the correlation between the age (in months) of which CMF products are covered under the national legal measures and per capita consumption across the four categories: Standard, follow-on, toddler, and special formulas.

For standard, follow-on, and special formulas, which are CMFs that are usually marketed from birth to 12 or 24 months (depending on the brand), an inverse correlation was evident. Countries with a higher age coverage in the scope of their legal measures (Vietnam up to 24 months and the Philippines up to 36 months), which also means stronger overall Code implementation, had lower per capita consumption in standard formula (Vietnam at 8.4 kg and Philippines at 9.0 kg), follow-on formula (Vietnam at 15.8 kg and Philippines at 10.8 kg), and special formula (Vietnam with no data and Philippines at 1.5 kg).

Those with unspecified age coverage or no legal measures in 2020 (thus no legally binding age coverage), namely Australia, Japan, Malaysia, South Korea, and New Zealand, exhibited higher per capita consumption of standard formula (Australia at 53.7 kg, Japan at 32.6 kg, New Zealand at 30.5 kg, and South Korea at 29.7 kg); as well as follow-on formula (Australia at 46.0 kg, New Zealand at 30.5 kg, and South Korea at 24.1 kg).

The correlation (*R*^2^) was 0.21 for standard, 0.31 for follow-on formula, 0.18 for special formula. For toddler formula, the correlation (*R*^2^) was about zero, meaning there is no relationship found between age coverage of CMF in national measures and per capita consumption (Figure 3).

### 3.6 Correlations between maternity protection and per capita consumption of CMF

**Figure 5:**
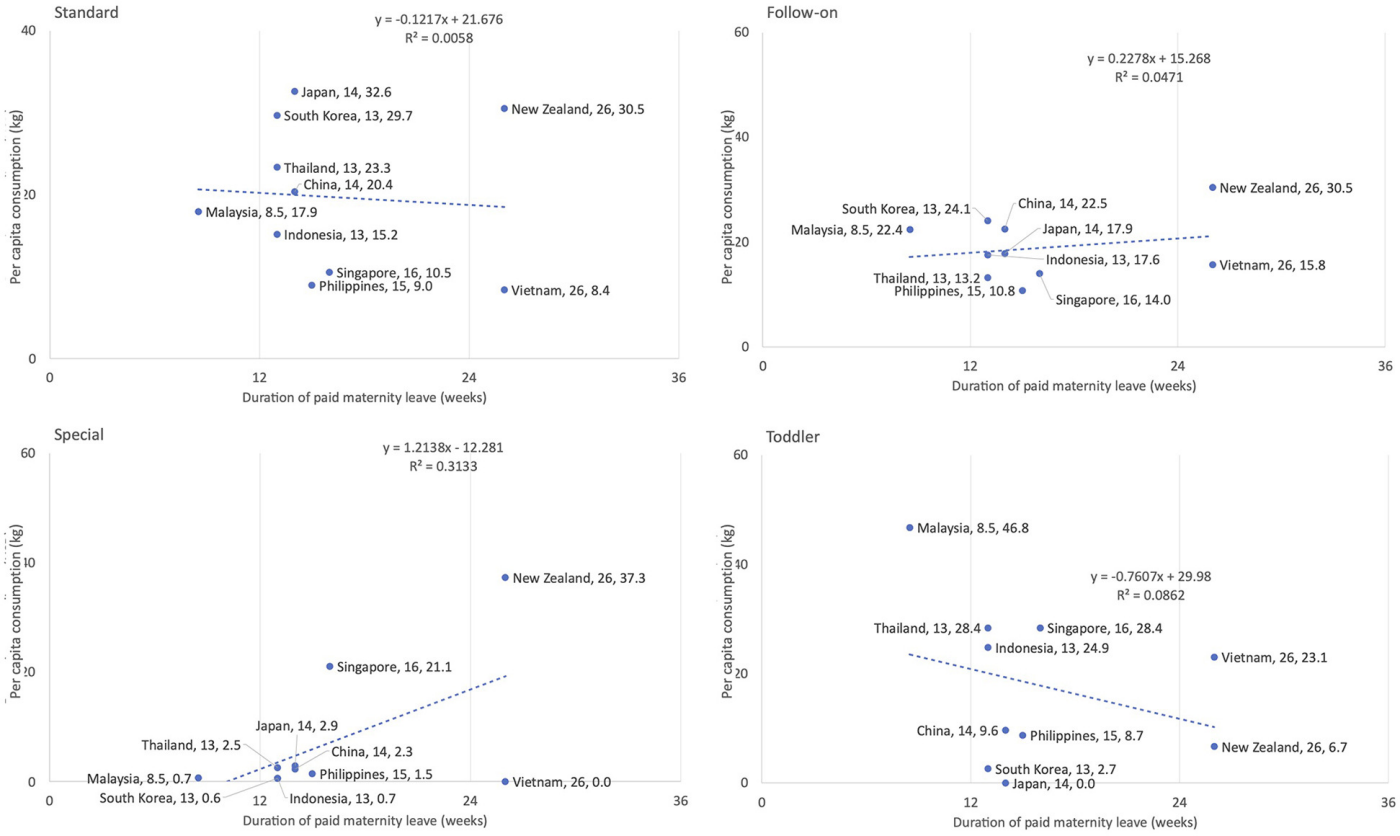
Association between the duration of paid maternity leave and per capita consumption of commercial milk formula in 2019 in East Asia Pacific region by type and country.

Figure 5 shows the correlations between the duration of paid maternity leave (in weeks) and per capita consumption on CMF across four CMF categories: Standard, follow-on, toddler, and special formulas. Overall, the correlations were inconsistent and varied by formula type. The trends for standard and follow-on formulas showed weak correlations (*R*^2^ = 0.0058 and 0.047, respectively), suggesting little to no relationship between paid maternity leave duration and CMF consumption. However, for special formulas, there was a positive correlation (*R*^2^ = 0.31), where longer paid maternity leave was associated with higher consumption. In contrast, for the toddler formula category, a slight negative correlation was observed (*R*^2^ = 0.086), indicating that longer paid maternity leave might slightly reduce consumption.

## 4 Discussion

### 4.1 Code implementation varies across the region

Strengthening the implementation of the Code in full through legal measures must be a public health priority for all countries. In the EAP region and globally, there is a substantial lag. A key contributor to this lag is the interference from CMF industry in the development of effective legislation, monitoring and enforcement of legal measures on the Code (19, 27, 35, 53). As of 2024, 146 out of 194 countries that are WHO Member States have adopted Code-related legal measures, but only 33 of them have legal measures that are substantially aligned with the Code (53). When the World Health Assembly adopted the Code through WHA Resolution 34.22 in 1981, it emphasises that the adoption of and adherence to the Code (which later includes relevant WHA resolutions) at the national level is a minimum requirement. Governments are encouraged to adopt additional or stronger provisions than those set out in the Code. The Code must be translated into suitable legal measures as appropriate to the legislative framework of the implementing country for it to take legal effect at the national level. For most countries in this study, there are still substantial gaps between national measures and the Code, which are often exploited by companies (3, 13).

In 2020, only six out of the 11 countries adopted the Code through legal measures. In 2024, three more countries, Australia, New Zealand, and Singapore, have adopted some Code-related legal measures. However, marketing of CMF in Australia and New Zealand is still largely addressed by voluntary measures (53). Malaysia and Japan, both without legal measures, are managing restrictions on marketing through voluntary measures with no enforcement mechanisms. Globally, there are 48 out of the 194 WHO Member States with no legal measures and largely relying on voluntary or industry measures to regulate marketing practices. These voluntary measures or agreements are not legally binding and are favoured by industry as they usually allow industry to influence the rules to ensure there are only minimal restrictions. These measures have proven to be ineffective as they do not have any mechanisms to compel compliance and there is no legal sanction attached to the breaches (13, 23). For instance, in Australia, the Australian Competition and Consumer Commission (ACCC) has recently denied the authorization for continued implementation of the Marketing in Australia of Infant Formula: Manufacturers and Importers Agreement (commonly known as the MAIF agreement), recommending the government to consider adopting legally-binding marketing restrictions based on concerns about the effectiveness of its voluntary nature, limited scope, and inability to capture the breadth of modern digital marketing methods (57).

Before the adoption of the WHA Resolution 69.9 (including the 2016 Guidance) which clarifies that the scope of CMF should be covered up to 36 months (including toddler formula), CMF industry frequently argued that the Code covers only standard formula (26). Out of the 11 countries, only the Philippines and Thailand cover CMF products up to 36 months. When national Code regulations fall short of covering CMF products up to the age of 36 months, it leaves gaps for companies to promote their follow-on and toddler formulas. Often the branding and labelling of the products not covered by the scope are deliberately and remarkably similar to that of their standard formula for the purpose of cross-promotion (13, 27, 58), undermining both exclusive and continued breastfeeding.

### 4.2 Correlations found between implementation of Code provisions as legal measures and CMF consumption

CMF sales volumes sales and per-capita consumption were high and in increased trend in most countries in the region. These CMF consumption figures in the EAP region surpass the global average and that of most other regions, except for certain categories in Western Europe and North America. While the EAP region demonstrated lower per capita consumption on standard, follow-on, and special formulas compared to Western Europe, it reports higher consumption on toddler formula. Similarly, compared to North America, the EAP region shows lower per capita consumption on standard and special formulas but higher consumption on follow-on and toddler formulas (37). Moreover, while Western Europe and North America exhibit steady or decreasing trends in CMF consumption, the EAP region demonstrates increasing trends across all CMF categories (37). The increases in CMF consumption are concerning as the expenses on these products also create economic burden on countries as well as individual households (5, 59).

The importance of Code implementation at national level can be inferred from the inverse correlations between the level of Code implementation and consumption on CMF. Where there were stricter regulations on marketing, the per capita CMF consumption tended to decrease. The Philippines, with highest score on Code implementation, showed the lowest per capita CMF consumption. As the main function of the Code is to restrict marketing, the findings also suggest that where Code implementation was weak and hence marketing was under little or no restrictions, CMF consumption tended to be higher, as seen in Australia, New Zealand, and Malaysia.

The Global Strategy on Infant and Young Child Feeding, endorsed by WHA Resolution 55.25, states that infants should be exclusively breastfed for the first six months of life to achieve optimal growth, development and health. Thereafter, to meet their evolving nutritional requirements, infants should receive nutritionally adequate and safe complementary foods while breastfeeding continues for up to two years and beyond (23). The 2016 Guidance reinforces this recommendation by stipulating that marketing of CMF should be restricted to at least 36 months. Out of the countries with legal measures, only Thailand and the Philippines covered CMF products up to 36 months. The observed inverse correlations between the age of which CMF products are covered and CMF consumption speak to the importance of ensuring the scope of CMF products covered is at a minimum of 36 months to protect both exclusive and continued breastfeeding.

For the toddler formula category, the lack of correlation with Code implementation could possibly be related to the overall weak or lack of regulation of marketing of toddler formula (marketed from 12 to 36 months) in most of the 11 countries. Only two countries had legal measures covering CMF products up to 36 months. The lack of regulation means companies are more likely to promote this product, which is potentially contributing to the rising consumption of toddler formula in ten out of the 11 countries in this region. This situation is consistently observed globally, as only 38 countries have measures clearly covering the full breadth of CMF up to 36 months of age (19, 53). This is especially problematic as this product is high in sugar content and addictive. Toddler formula has been deemed as unnecessary for proper growth and development by WHO and it is not recommended for young children by public health authorities such as the American Academy of Pediatrics (60). The regulation gap is also commonly exploited for cross-promotion of standard formula (discussed above in Section 4.1) that undermines exclusive breastfeeding.

The correlations found are consistent with the non-statistical observation from an earlier study (50) that found standard formula, a CMF product marketed from birth and most commonly covered in national legal measures, had consistently lower sales value and volume compared to other CMF products marketed for older infants and young children. This study has filled the previous research gap by using simple linear regression analyses and has provided some preliminary statistical patterns to support the messages from a number of landmark studies that linked together the alarming surge in CMF sales around the globe, increasingly multifaceted and well-resourced marketing playbook and its influence on families, health professionals, science, and policymaking, and insufficient Code implementation in countries (13, 19, 33, 36, 49, 61). Though with limitations (addressed in Section 4.4), future studies can build upon these findings to develop sound hypotheses.

### 4.3 Maternity protection and CMF consumption: room for further exploration

Even though the findings from the linear regression analysis of this initial exploratory study, which included a limited number of countries from one region, indicated no clear correlations between paid maternity leave duration and CMF consumption, the observations however suggest that the five countries (i.e., China, Japan, the Philippines, South Korea, Vietnam) with a relatively lower annual per capita CMF consumption provide paid breastfeeding breaks of at least 40 min per day when mothers return from maternity leave.

Toddler formula emerged as the highest-value category compared to other CMF categories in countries Indonesia, Thailand and Malaysia, maternity protection was not aligned with the ILO MPC. Australia, with the second highest standard and follow-on formula sales per child in 2019, has one of the best-paid maternity leave policies but no legal measures to give effect to the Code (in 2019), and still largely addresses marketing through voluntary measures.

Even though the findings from this exploratory study do not demonstrate a clear relationship between paid maternity leave and CMF consumption, there is convincing evidence from existing studies highlighting that improved access to maternity protection (especially paid maternity leave and workplace breastfeeding support) as well as paid family leave are associated with increased breastfeeding rates (12, 40, 62). This would also imply opposing implications for CMF consumption. Furthermore, for vulnerable groups of workers, such as those employed informally, while there are documented challenges with extending social protection to informal workers (63), for paid maternity leave, this should be considered a “triple duty action” as it forms a component of access to labour rights, and social protection but also has public health advantages, as improved paid maternity leave in LMIC is associated with increased breastfeeding rates (40).

The findings should not rule out the possibility that other components of maternity protection policy (e.g., paid breastfeeding breaks, informal sector coverage), in combination with paid maternity leave, as having potential correlations with CMF consumption. It would be beneficial to conduct a more systematic and in-depth analysis of associations between various components of maternity protection and CMF consumption across different contexts and a greater number of countries and regions, which may provide different results.

### 4.4 Study limitations

A scoring algorithm was used in the Code status report to assess itemised content contained in national legal measures. While the total scores offer a broad-stroke overview of the implementation of Code provisions in national measures, they cannot adequately reflect the quality of restrictions as the legal provisions are often couched in complicated legalese. Certain specificities of Code provisions cannot be adequately assessed by the scoring algorithm. For instance, the algorithm does not consider whether monitoring and enforcement are taking place and whether they are integrated into existing national inspection or regulatory systems. It also does not have any mechanism to measure the extent of restrictions of certain marketing practices outlined in the Code (e.g., whether a certain practice is restricted unless with permission from the government or a complete ban). Moreover, the algorithm also does not indicate whether the national legal measures are sufficient in addressing marketing on digital platforms, where pervasive promotion has rapidly increased in recent years.

Regarding consumption data, even though the products covered by the Code (and relevant WHA resolutions) include CMF (up to 36 months), foods for infants and young children (six to 36 months), and feeding bottles and teats, and that it is also important to assess the correlation between Code implementation and consumption of foods for infants and young children and feeding bottles and teats, this study could only focus on CMF consumption as limited by access to data (data accessible by the authors was limited to CMF products marketed up to 36 months).

Even though this is the first known study to conduct statistical analyses between national Code implementation status (using Code implementation scores) and CMF consumption, the correlations are only meant to be used to explore potential patterns. Further investigations with stronger data and analysis are needed to develop hypotheses. Other factors believed to influence breastfeeding and consumption on CMF, such as national income, household income, women's labour force participation rate, aging population in the region, birth rates, access to breastfeeding support and knowledge, and cultural and social beliefs, were not accounted for. Another key factor that is not examined in this study is the status of Code monitoring and enforcement. Often, even when there are laws to give effect to the Code, the monitoring and enforcement are lagging (27, 52, 53). Enforcement can only take place in countries with legal measures, and effective enforcement is dependent upon a functional monitoring system and enforcement mechanism, appropriate penalties, and an intact legal infrastructure. Overall, monitoring and enforcement of Code measures in most countries are inconsistent and lagging, and it is extremely difficult to gather information on enforcement [(64) in press].

The correlation between maternity protection and CMF consumption was only tested using availability of paid maternity leave duration. The existence of national paid maternity leave policies does not guarantee that all women can access paid maternity leave. There are a number of factors that affect women's access to paid maternity leave even when a country has adopted relevant policies. Many factors reduce the accessibility of paid maternity leave, including inadequate payment value, insufficient coverage for all types of workers, complicated implementation frameworks, and fragmented policies (46, 65). Other forms of maternity protection, including lactation breaks at work, were not included in the regression analysis. Future research can include empirical analysis of these other factors, especially since this is the first time that correlations between paid maternity leave and CMF consumption have been explored statistically.

## 5 Conclusion and recommendations

To conclude, Code implementation in the EAP region is still inadequate when compared to the Code and relevant WHA resolutions as a minimum benchmark, with a few countries having no legal measures and relying on ineffective voluntary measures. Overall CMF consumption is high in the region compared to global and other regions’ average, and the region contributed over half of the global toddler formula consumption - a product that is unsuitable for consumption young children yet not included in the scope of national measures in many countries in the region and around the world. Code implementation (among other possible factors) and specifically the scope of CMF products included in legal measures have shown to have inverse correlations with CMF consumption, suggesting where marketing was under little to no restriction, CMF consumption tended to increase, and vice versa. These correlation findings, though with limitations, can be built upon in future studies. Though there are no clear correlations demonstrated between paid maternity leave and CMF consumption, the findings still suggested the importance of adopting comprehensive maternity protection policy and its possible role in reducing CMF consumption. However, the adoption of policies should not hinge purely upon their correlations with CMF consumption, as both the Code and maternity protection have broader public health and human rights relevance.

It is important for countries to close the gaps between the Code (including relevant WHA resolutions) and national measures through adopting legal measures, in particular bringing the age range of the scope of CMF in national measures on par with the 2016 Guidance as a minimum (up to 36 months). Countries should also ensure that legal measures and monitoring and enforcement mechanisms address the marketing tactics made possible through digital platforms. As adopting laws usually takes times but given the rising consumption of toddler formula in the region, raising awareness on the relevant health risks and the WHO recommendation against its use can be an urgent action. Findings on maternity protection indicate the need to strengthen comprehensive maternity protection policy, including sufficient payment while on maternity leave, increased coverage to all types of workers and reduced administrative barriers to access. The findings have pointed to the importance of intersectoral policy interventions, including a combination of Code implementation and comprehensive and accessible maternity protection, together with the other actions addressed in the seven key policy and programme actions outlined by the Global Breastfeeding Collectives.

Code implementation and maternity protection are two out of seven critical policy priorities identified by the Global Breastfeeding Collective to protect breastfeeding, which is crucial in improving child malnutrition, ensuring normative child development, mitigating food insecurity, contributing to the national and global economy, and reducing carbon and water footprints. Given the momentum gained in the recent adoption of a landmark WHA resolution that urges countries to strengthen marketing restrictions of CMF and related products, including digital marketing, it is timely to reinvigorate the commitment and collaboration from governments and civil society to achieve the World Health Assembly's 2025 global breastfeeding target – without any delay.

## Data Availability

The original contributions presented in the study are included in the article/Supplementary Material, further inquiries can be directed to the corresponding authors.
