# Supplementary material for: Implementation of the International Code of Marketing of Breast-milk Substitutes and maternity protection: correlations with commercial milk formula consumption in East Asia and the Pacific
Source: Front Pediatr. 2025 Jul 9;13:1553599. doi: 10.3389/fped.2025.1553599 (PMC12283749; doi:10.3389/fped.2025.1553599)
Supplement: Supplementary file 1 [file Table1.docx]

**Table 2.** Relevant WHA Resolutions: Key Points

| **Year** | **Resolution** | **Key points** |
| --- | --- | --- |
| 1981 | WHA 34.22 | - Stresses that adoption and adherence to the Code is a minimum requirement. Member States are urged to implement the Code into national legislation, regulations and other suitable measures. |
| 1982 | WHA35.26 | - Recognizes that commercial promotion of breastmilk substitutes contributes to an increase in artificial feeding and calls for renewed attention to implement and monitor the Code at national and international levels. |
| 1984 | WHA37.30 | - Requests that the Director General work with Member States to implement and monitor the Code and to examine the promotion and use of foods unsuitable for infant and young child feeding. |
| 1986 | WHA39.28 | - Urges Member States to ensure that the small amounts of breastmilk substitutes needed for a minority of infants are made available through normal procurement channels and not through free or subsidized supplies. - Directs attention of Member States to the following: 1. Any food or drink given before complementary feeding is nutritionally required may interfere with breastfeeding and therefore should neither be promoted nor encouraged for use by infants during this period; 2. The practice of providing infants with follow up milks is “not necessary". |
| 1988 | WHA41.11 | - Requests the Director General to provide legal and technical assistance to Member States in drafting or implementing the Code into national measures. |
| 1990 | WHA43.3 | - Highlights the WHO/UNICEF statement on “protection, promoting and supporting breastfeeding: the special role of maternity services” which led to the Baby-Friendly Hospital Initiative in 1992. - Urges Member States to ensure that the principles and aim of the Code are given full expression in national health and nutrition policy and action. |
| 1994 | WHA47.5 | - Reiterates earlier calls in 1986, 1990 and 1992 to end “free or low cost supplies” and extends the ban to all parts of the health care system. - Provides guidelines on donation of breastmilk substitutes in emergencies. |
| 1996 | WHA49.15 | - Calls on Member States to ensure that: 1. complementary foods are not marketed for or used to undermine exclusive and sustained breastfeeding; 2. financial support to health professionals does not create conflicts of interests; 3. Code monitoring is carried out in an independent, transparent manner free from commercial interest. |
| 2001 | WHA 54.2 | - Sets global recommendation of “6 months” exclusive breastfeeding, with safe and appropriate complementary foods and continued breastfeeding for up to two years or beyond. |
| 2002 | WHA55.25 | - Endorses the Global Strategy on Infant and Young Child Feeding which confines the baby food manufacturers and distributors’ role to: 1. ensuring quality of their products; 2. complying with the Code and subsequent WHA resolutions, as well as national measures. - Recognizes the role of optimal infant feeding to reduce the risk of obesity. Alerts that micronutrient interventions should not undermine exclusive breastfeeding. |
| 2005 | WHA58.32 | - Asks Member States to: 1. ensure that nutrition and health claims for breastmilk substitutes are not permitted unless national/regional legislation allows; 2. be aware of the risks of intrinsic contamination of powdered infant formulas and to ensure this information be conveyed through label warnings; 3. ensure that financial support and other incentives for programs and health professionals working in infant and young child health do not create conflicts of interest. |
| 2006 | WHA59.11 | - Member States to make sure the response to the HIV pandemic does not include non-Code compliant donations of breastmilk substitutes or the promotion thereof. |
| 2006 | WHA59.21 | - Commemorates the 25th anniversary of the adoption of the Code; welcomes the 2005 Innocenti Declaration and asks WHO to mobilize technical support for Code implementation and monitoring. |
| 2008 | WHA61.20 | - Urges Member States to: 1. scale up efforts to monitor and enforce national measures and to avoid conflicts of interest; 2. investigate the safe use of donor milk through human milk banks for vulnerable infants, mindful of national laws, cultural and religious beliefs. |
| 2010 | WHA63.23 | - Urges Member States to: 1. strengthen implementation of the Code and resolutions, the Global Strategy on Infant and Young Child Feeding, the Baby-Friendly Hospital Initiative, the Operational Guidance for Emergency Relief Staff; 2. end all forms of inappropriate promotion of foods for infants and young children and that nutrition and health claims should not be permitted on these foods. - Urges corporations to comply fully with responsibilities under the Code and resolutions. |
| 2012 | WHA65.6 | - Urges Member States to put into practice the comprehensive implementation plan on maternal, infant and young child nutrition, including: 1. developing or strengthening legislative, regulatory or other measures to control the marketing of breastmilk substitutes; 2. establishing adequate mechanisms to safeguard against potential conflicts of interest in nutrition action. - Requests the Director General to: 1. provide clarification and guidance on the inappropriate promotion of foods for infants and young children as mentioned in WHA63.23; 2. develop processes and tools to safeguard against possible conflicts of interest in policy development and implementation of nutrition programs. |
| 2014 | WHA67(9) | - Infant and Young Child Nutrition (MIYCN) Plan which includes increasing the rate of exclusive breastfeeding to at least 50% by 2025 as a global target. The indicator for regulation of marketing is the number of countries with legislation or regulations fully implementing the Code and Resolutions. |
| 2016 | WHA69.9 | - This Resolution welcomes the WHO Guidance on ending the inappropriate promotion of foods for infants and young children. It calls upon 1. Member States to take all necessary measures to implement the Guidance 2. Manufacturers and distributors of foods for infants and young children to adhere to the Guidance. The Guidance clarified that follow-up milks and growing up milks are covered by the Code and should be treated as such when implementing the Code. The Guidance also recommends that there should be no cross-promotion to promote breastmilk substitutes via the promotion of foods for infants and young children. |
| 2018 | WHA71.9 | - This Resolution urges Member States to: 1. reinvigorate the Baby-friendly Hospital Initiative and the full integration of the revised 10 Steps to Successful Breastfeeding which incorporates Code compliance in Step 1; 2. take all necessary measures to implement recommendations to end the inappropriate promotion of foods for infants and young children. |
| 2020 | WHA73.26 | - Requests the Director-General to review current evidence and prepare a comprehensive report on the scope and impact of digital marketing strategies for the promotion of breast-milk substitutes to the 75th World Health Assembly in 2022. |
| 2022 | WHA75.21 | - Requests the Director-General to develop guidance for Member States on regulatory measures to restrict the digital marketing of breastmilk substitutes, to ensure existing and new regulations designed to implement the International Code of Marketing Breast-milk Substitutes and relevant WHA resolutions adequately address digital marketing practices, and report progress in the 77th WHA 2024. |
| 2024 | WHA 78.18 | - The Resolution highlights digital marketing of breastmilk substitutes and related products, including influencer marketing, has become the dominant form of marketing in many countries, and it may not be effectively regulated. The Resolutions urges Member States to implement the recommendations of the Guidance on regulatory measures aimed at restricting digital marketing of breast-milk substitutes into national regulatory frameworks and monitor its application, and for the Director-General to provide support to Member States. |
