# Supplementary material for: Implementation of the International Code of Marketing of Breast-milk Substitutes and maternity protection: correlations with commercial milk formula consumption in East Asia and the Pacific
Source: Front Pediatr. 2025 Jul 9;13:1553599. doi: 10.3389/fped.2025.1553599 (PMC12283749; doi:10.3389/fped.2025.1553599)
Supplement: Supplementary file 2 [file Table2.docx]

**Table 1.** Summary of the International Code of Marketing of Breast-milk Substitutes

| Aim | To contribute to the provision of safe and adequate nutrition for infants by the protection and promotion of breastfeeding and the proper use of breastmilk substitutes, when these are necessary, on the basis of adequate information and through appropriate marketing and distribution. |
| --- | --- |
| Scope | Applies to breastmilk substitutes* ^1^ or any food being marketed or otherwise represented as a partial or total replacement for breastmilk. This includes:   - Infant formula - Follow-up formula (sometimes referred to as ‘follow-on milk1) * - Growing-up milk * - Any other milk for children 0 < 36 months * - Any other food or liquid (such as cereal, jarred food, infant tea, juice and mineral water) that is represented as suitable to be fed to infants less than six months of age. *   The International Code also applies to feeding bottles and teats. |
| Promotion | No advertising or promotion of above products to the public. No nutrition or health claims on products. *^ ^2^ |
| Samples | No free samples to mothers, their families, or health care workers. |
| Health care facilities | No promotion of products, i.e., no product displays, posters, calendars, or distribution of promotional materials. No mothercraft nurses or similar corporation-paid personnel. |
| Health care workers | No gifts or samples to health care workers. Financial support and incentives should not create conflicts of interest. ^ ^3^ |
| Supplies | No free or low-cost supplies of breastmilk substitutes to any part of the health care system. ^ ^4^ |
| Information | Information and education materials must explain the benefits of breastfeeding, the health hazards associated with bottle-feeding, and the costs of using infant formula. Product information must be factual and scientific. Governments to avoid conflicts of interest so materials under infant and young child programs should not be sponsored by manufacturers and distributors. ^ ^5^ |
| Labels | Product labels must clearly state the superiority of breastfeeding, the need for the advice of a health care worker, and a warning about health hazards. No pictures of infants, other pictures, or text idealizing the use of infant formula. Labels must contain the warning that powdered infant formula may contain pathogenic microorganisms and must be prepared and used appropriately. ^ ^5^ Labels on complementary foods should not cross-promote breastmilk substitutes, should not promote bottle-feeding, and should state the importance of continued breastfeeding. ^ ^6^ |
| Quality | Unsuitable products, such as sweetened condensed milk, should not be promoted for babies. All products should be of a high quality (Codex Alimentarius Standards) and take account of the climatic and storage conditions of the country where they are used. |

(*) denotes products and definitions which are clarified by the WHO Guidance on ending the inappropriate promotion of foods for infants and young children Guidance A69/7 Add.1
which was welcomed by WHA Resolution 69.9 [2016].
(^) denotes that Code provisions have been clarified and extended by subsequent World Health Assembly Resolutions which are summarized in Annex B.

^1^ WHA49.15 [1996], WHA54.2 [2001] & WHA63.23 [2010]

^2^ WHA58.32 [2005] & WHA63.23 [2010]
^3^ WHA49.15 [1996] & WHA58.32 [2005]
^4^ WHA47.5 [1994] v. WHA58.32 [2005]

^5^ WHA58.32 [2005]

^6^ A69/7 Add.1
